# Feeding foliar nano-selenium biofortified panax notoginseng could reduce the occurrence of glycolipid metabolism disorder in mice caused by high-fat diets

**DOI:** 10.3389/fnut.2022.973027

**Published:** 2022-08-24

**Authors:** Qinyong Dong, Sen Yan, Dong Li, Chunran Zhou, Sinuo Tian, Yu Wang, Peijuan Miao, Wentao Zhu, Shusheng Zhu, Canping Pan

**Affiliations:** ^1^Department of Applied Chemistry, College of Science, Innovation Center of Pesticide Research, China Agricultural University, Beijing, China; ^2^State Key Laboratory for Conservation and Utilization of Bio-Resources in Yunnan, College of Plant Protection, National Engineering Research Center for Applied Technology of Agricultural Biodiversity, Yunnan Agricultural University, Kunming, China

**Keywords:** nano-selenium, high-fat diet, Panax notoginseng, glycolipid metabolism, gut microbiota

## Abstract

Nano-selenium (nano-Se) has been extensively explored as a biostimulant for improving the quality of grain crops. However, there are few reports about the effect on the medicinal components of Chinese herbal medicine cultured with nano-Se. Here, we sprayed nano-Se during the cultivation of Panax notoginseng (SePN), and measured the changes of medicinal components compared with conventional Panax notoginseng (PN). Furthermore, we identified a more pronounced effect of SePN on reducing obesity in animals compared with PN. By measuring antioxidant capacity, histopathology, gene expression related to glycolipid metabolism, and gut microbiota composition, we propose a potential mechanism for SePN to improve animal health. Compared with the control groups, foliar spraying of nano-Se increased saponins contents (Rb2, Rb3, Rc, F2, Rb2, and Rf) in the roots of Panax notoginseng, the content of Rb2 increased by 3.9 times particularly. Interestingly, animal studies indicated that taking selenium-rich Panax notoginseng (SePN) can further ameliorate liver antioxidation (SOD, MDA, and GSH) and enzyme activities involved in glycolipid metabolism (ATGL and PFK). It also relieved inflammation and regulated the expression of genes (*MCAD, PPAR-*α, and *PCSK9*) related to fatty acid oxidation. The abundance ratio of *Firmicutes/Bacteroides* and beneficial bacteria abundance (*Bifidobacterium, Butyricimonas*, and *Parasutterella*) in gut microbiota were improved relative to the control. In summary, the application of nano-Se on PN may effectively raise the content of Panax notoginseng saponins (PNS) and immensely lower the risk of metabolic disorders of glycolipids.

## Introduction

At present, more than 600 million individuals are obese, accounting for 39% of the world's adult population (1). Obesity raises the risk of a variety of diseases, including insulin resistance, cardiovascular disease, liver disease, and cancer (2). Obesity is frequently assumed to be caused by a high-fat and high-calorie food supply (3). People progressively recognize the harm of the high-fat diet (HFD) and the importance of a scientific diet, therefore they focus on enhancing bodily function through the use of functional foods (4, 5). Currently, Chinese herbs such as Panax ginseng, Panax notoginseng, and Chinese yam have been experimentally validated for reducing the risk of cardiovascular disease in animal studies (6–8).

Panax notoginseng (PN) is a perennial Chinese herbal medicine with Panax notoginseng saponins (PNS) as its principal functional component. PNS are useful for hemostasis, anti-inflammation, analgesia, and the prevention of cardio-cerebrovascular diseases (9). PNS can dramatically up-regulate the expression of fatty acid β-oxidation related factor mRNA (*MCAD, LCAD*) to intervene with cardiomyocyte hypertrophy by regulating energy metabolism in the field by lowering blood sugar and blood lipids (10). PNS has also been demonstrated to boost the expression of *CPT-1A* in T cells, increasing their fatty acid oxidation metabolism, and promoting the differentiation of mice ${\text{CD}}_{4}^{+}$T cells into Treg cells (11). In a nutshell, PN which can reduce blood sugar and blood lipid levels should be investigated further. However, Chinese herbal medicine is vulnerable to abiotic (heavy metals, drought, flooding, climate change, pesticides) and biotic stress (insects and pathogenic bacteria), which affects the composition and production of medicinal components (12).

Exogenous hormones such as jasmonic acid, salicylic acid, and melatonin have been shown in several studies to effectively alleviate the deleterious effects of biotic and abiotic stress on PN, as well as boost PN biomass and antioxidant capacity (13–15). However, there are few investigations on the intervention of Se with a strong antioxidant capacity of the medicinal components of the PN. Se exerts biological benefits as a component of the antioxidant enzyme glutathione peroxidase (GPX) (16). Foliar Se treatment reduced oxidative stress damage while also increasing grain yield and Se contents (17). Maassoumi et al. (19) found that Se can up-regulate triterpenoid saponins, soluble sugar, amino acids, and exopolysaccharides contents in Astragalus (18). Compared with inorganic and organic Se, nano-Se has high bio-availability, superior stability, minimal toxicity, and great free radical scavenging capabilities. Studies showed that nano-Se may be utilized as biofortifiers and stimulators, and its effect on plant antioxidant metabolism was related to primary and secondary metabolites (20). Our previous studies demonstrated that foliar application of nano-Se regulated hormone pathway, phenylpropane pathway, volatile organic compounds, antioxidants enzymes, and secondary metabolites to strengthen the quality and resistance in various crops (21–23). Medical studies revealed that the protective mechanism of Se on the human body includes inhibition of oxidative stress, endothelial dysfunction, protection of vascular cells from apoptosis, calcification, and regulation of inflammation (24). Long-term Se deficiency in the human body can harm the cardiovascular system and lead to myocardial infarction (25). However, few researchers have studied the mechanism of how nano-Se improves the quality of traditional Chinese medicine PN and the level of glycolipid metabolism after ingesting nano-Se-cultivated PN.

Hence, the saponins contents, physiological and biochemical indexes, antioxidant capacities, enzymes activities, gene expression, pathological analysis, and gut microbiota connected with glycolipid metabolism were targeted determination to explore nano-Se foliar applications in PN acts the effect on glycolipid metabolism in mice.

## Materials and methods

### Synthesis and characterization of nano-se

The synthesis method refers to the previous research (26). The 1% chitosan solution was prepared for the pre-solution, and then 20 mM of selenium dioxide solution (i.e., selenite solution) was slowly added to 20 mL of the pre-solution. The nanoscale dispersed selenite colloidal solution was obtained by continuous stirring at 500 rpm and 25°C. Slowly add 4 ml 1% ascorbic acid solution and stir continuously at 25°C at the speed of 500 rpm for 3 h until the solution color changes to transparent red, which means that the synthesis of nanometer selenium is over. For characterization data, please refer to our previous article (22).

### Cultivation and processing of SePN

The SePN plants were grown in Kunming, Yunnan Province, China. The cultivation method is to select 1-year-old PN seedlings. Nano-Se is sprayed once a month from May to November, and root samples of PN were collected in December. According to the recommended dosage of PN in Chinese Pharmacopeia and the rule of body surface area, the roots PN were ground into powder by a mill and added into HFD to make the mass fraction of PN 6 g/Kg. All reagents needed in the experiment were purchased from commercial channels.

### Sample preparation of PN

The 0.1 g mashed PN powder was weighed in a 2 ml centrifuge tube. The saponin was extracted by adding the 1 ml solution (70:30% V/V, methanol/water). The mixture was shaken for the 2 min on the VX-III multi-tube eddy current meter (Beijing Tajin Science and Technology, Beijing, China) and centrifuged for the 5 min at 10,000 rpm. Then, 1 ml supernatant was transferred to a 2 ml centrifuge tube containing 50 mg C18. The tube was vortexed for 2 min before being centrifuged at 10,000 rpm for 5 min. Finally, the supernatant was filtered through a 0.22 μm nylon filter and transferred into an autosampler glass vial for the saponin measurement.

### Relative quantification of saponins by HPLC–MS/MS

The HPLC–MS/MS system comprised an Agilent Series 1,290 ultra-performance liquid chromatography system and an Ultivo triple quadrupole mass spectrometer (Agilent Technologies, Palo Alto, CA, USA). The chromatographic separation was performed on a ZORBAX Eclipse Plus C18 chromatography column (2.1 × 50, 1.8 μm, Agilent) using a gradient elution of 0.1% formic acid in water (A) and acetonitrile (B) at a flow rate of 0.4 mL/min. The gradient profile was optimized as below, 0–10 min: 5–95% B, 10–12 min: 95–5% B, and 12–15 min: 5% B. The column temperature was maintained at 40 °C, and the injection volume was 2 μL. The composition changes of PNS are shown in Supplementary Table S1. The MRM parameters of each analytes are listed in Supplementary Table S2.

### Animals and treatment

Thirty 2-week-old C57 male mice were randomly divided into three groups with 10 mice in each group. They were placed in a stable environment with a temperature of 25 ± 2°C, and humidity of 50 ± 5% with 12 h light/12 h dark cycle. Mice were fed with HFD mixed with PN/SePN and free drinking water for 8 weeks. Body weight and food intake were recorded every 7 days. The mice were fasted for 1 day after collecting their fecal flora, and dissected after fasting to collect liver, heart and serum samples. These samples were collected and stored in −80°C. All experimental operations were approved by the independent Animal Ethical Committee of China Agricultural University.

### Biochemical parameters assay of serum

Serum biochemical indexes include aspartate aminotransferase (AST), alanine aminotransferase (ALT), triglyceride (TG), glucose levels (GLU), total cholesterol (TC), and high-density lipoprotein cholesterol (HDL-C). They were measured using their respective assay kits (Nanjing Jiancheng Bioengineering Institute, China).

### Biochemical indexes assay of liver

The detection of superoxide dismutase (SOD), malondialdehyde (MDA), and glutathione (GSH) can reflect the oxidative stress indexes of the liver. Phosphofructokinase (PFK), hydroxymethylglutaryl CoA reductase (HMG-CoAR), and adipose triglyceride lipase (ATGL) were detected to reflect the enzyme activity of liver lipid metabolism. These indexes were measured by using the test kit of Nanjing Jiancheng Bioengineering Research Institute (China).

### Histopathological analysis of liver and colon

A small piece of liver and colon tissue (about 50 mg) was fixed with a 4% formaldehyde solution. After being embedded in dehydrated paraffin, the tissue was cut into 5 mm slices and then stained with hematoxylin-eosin. Histopathological images were collected by Olympus BX51 imaging system (Olympus Corporation, Japan) and analyzed by Image pro plus 6.0 software (Media Cybernetics, USA).

### Total RNA extraction and reverse ranscription

Total RNA was extracted using Trizol-A+ reagent (Tiangen Biotech Co., LTD., Beijing, China). The total RNA was reverse transcribed into cDNA using the FastQuant RT kit (Tiangen Biotech Co., LTD., Beijing, China). All cDNA was stored at −20°C for further testing.

### RT-qPCR analysis

Analysis of reverse-transcribed data with Thermofisher 7,500 instrument. The mRNA levels of genes were quantified and normalized against the housekeeping gene β*-actin*, according to the 2^−ΔΔCT^ method (27). The sequence information of all primers is listed in Supplementary Table S3.

### Extraction and analysis of microbial DNA

Total genomic DNA samples were extracted using the OMEGA Soil DNA Kit (M5635-02) (Omega Bio-Tek, Norcross, GA, USA), and stored at −20°C before further analysis. PCR amplification of the bacterial 16S rRNA genes V3–V4 region was performed using the forward primer 338F (5′-ACTCCTACGGGAGGCAGCA-3′) and the reverse primer 806R (5′-GGACTACHVGGGTWTCTAAT-3′). Amplification system including 5×reaction buffer 5 μL, 5×GC buffer 5 μL, dNTP (2.5 mM) 2 μL, Forward primer (10 uM) 1 μL, Reverse primer (10 uM) 1 μL, DNA Template 2 μL, ddH_2_O 8.75 μL, Q5 DNA Polymerase 0.25 μL. Amplification parameters include initial denaturation 98°C 2 min, denaturation 98°C 15 s, annealing 55°C 30 s, extension 72°C 30 s, final extension 72°C 5 min, 10°C hold. 25-30 cycles dsDNA Assay Kit (Invitrogen, Carlsbad, CA, USA). PCR amplicons were purified and quantified with Vazyme VAHTSTM DNA Clean Beads (Vazyme, Nanjing, China) and quantified using the Quant-iT PicoGreen dsDNA Assay Kit (Invitrogen, Carlsbad, CA, USA), respectively. Amplicons were pooled in equal amounts, and pair-end 2×250 bp sequencing was performed using the Illlumina NovaSeq platform with NovaSeq 6000 SP Reagent Kit (500 cycles) at Shanghai Personal Biotechnology Co., Ltd (Shanghai, China).

### Statistical analyses

All results are represented as the mean ± SD. The graphical illustrations were processed by Origin64 and GraphPad Prism 9 (OriginLab (MA), GraphPad (CA), USA), and the statistical analyses were performed using SPSS v19.0 (IBM, USA). One-way ANOVA and Tukey were used to test the significant differences of variables among different groups. Significant differences in gut microbiota between treatments were analyzed using principal coordinates analysis (PCoA). QIIME2 (2019.4) software was used to analyze the taxonomic composition. Community composition difference was analyzed by the LEfSe test.

## Results

### Body weight, diet and organ weight of mice

**Figure 1:**
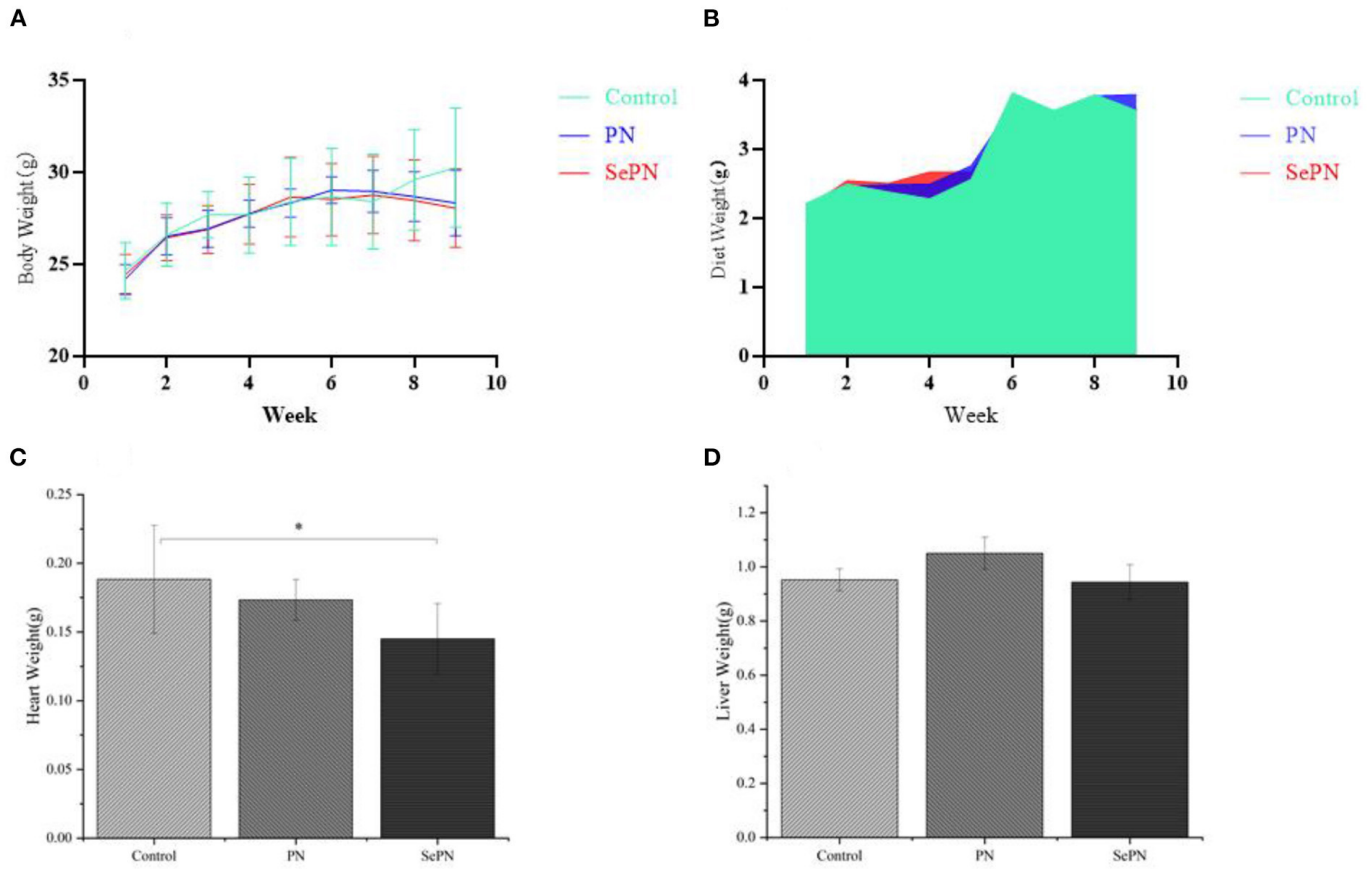
Body weight, diet and organ weight of mice. **(A)**. Body weight after 9 weeks of feeding high-fat diet (Control), Panax notoginseng (PN), and selenium-rich Panax notoginseng (SePN). **(B)**. Dietary intake within 9 weeks. **(C,D)**. Liver and heart weight after 9 weeks of feeding PN/SePN. Data are expressed as the mean ± SD. *P < 0.05 compared with the control group (*n* = 9).

Compared with the control group, there was a trend of weight loss during the 7th and 9th weeks and no alteration in daily food intake. There was no significant difference between the groups (Figures 1A,B). In addition, the heart weight of the mice in the SePN group decreased significantly (Figure 1C), but the liver weight did not change (Figure 1D).

### Glucolipid metabolism physiological indexes in serum of mice

**Figure 2:**
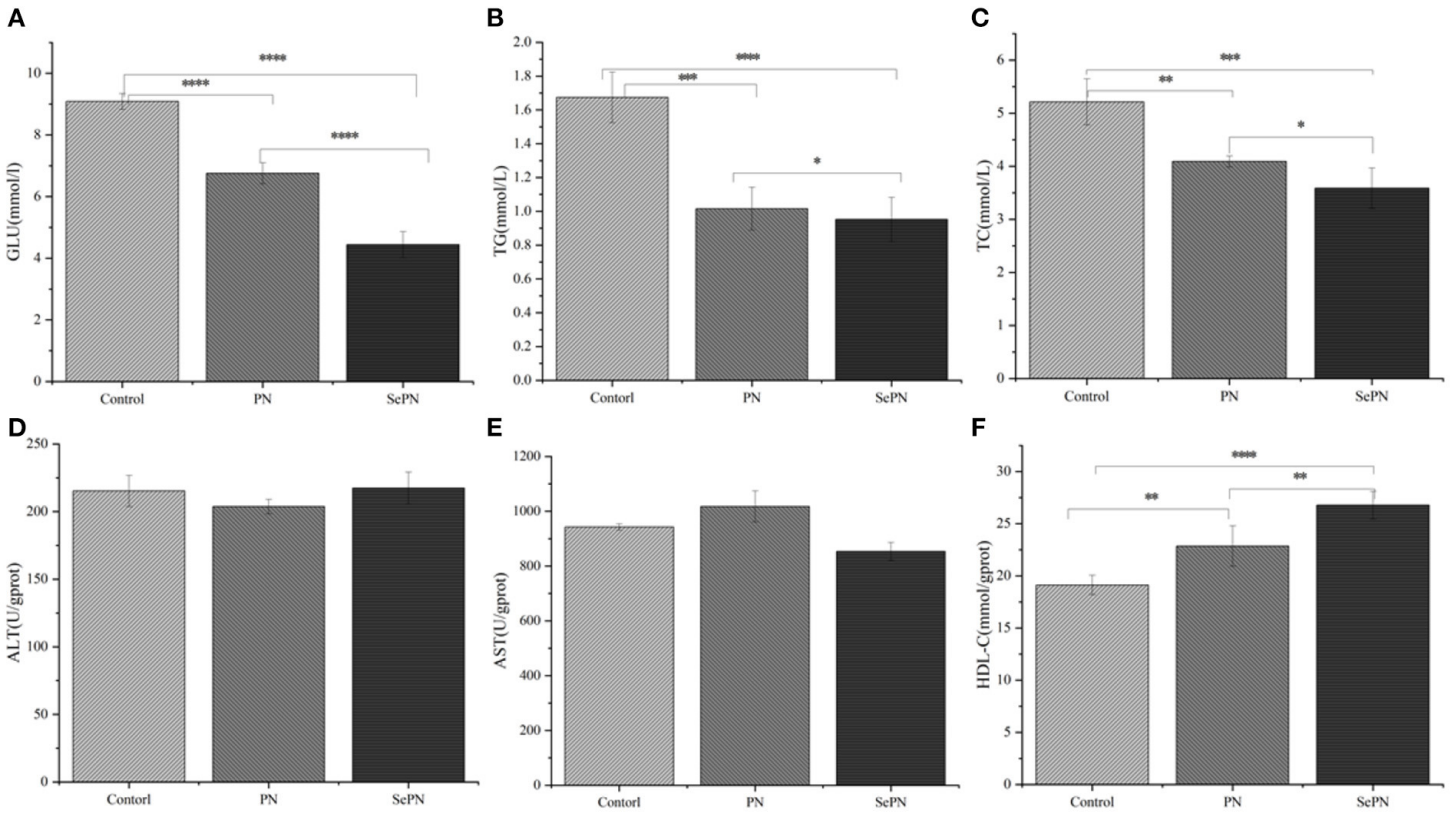
Glucolipid metabolism physiological indexes in serum of mice. **(A–C)**. The content of glucose (GLU), triglyceride (TG), and total cholesterol (TC) in the serum. **(D,E)**. The level of alanine aminotransferase (ALT) and aspartate aminotransferase (AST). **(F)**. High-density lipoprotein cholesterol (HDL-C). Data are expressed as the mean ± SD. *P < 0.05, **P < 0.01, ***P < 0.001, and ****P < 0.0001 compared with the control group (*n* = 5).

We detected the physiological and biochemical indexes related to glycolipid metabolism and found that ingesting SePN could significantly reduce the GLU content of mice compared with PN (Figure 2A). The same results were also reflected in physiological and biochemical indicators related to lipid metabolisms, such as TG and TC (Figures 2B,C). Furthermore, the treatment groups (PN, SePN) did not reduce the level of ALT and AST levels (Figures 2D,E). Taking SePN increased HDL-C contents in serum to a higher extent (Figure 2F).

### Antioxidant and enzymes activities of glucolipid metabolism in the liver

**Figure 3:**
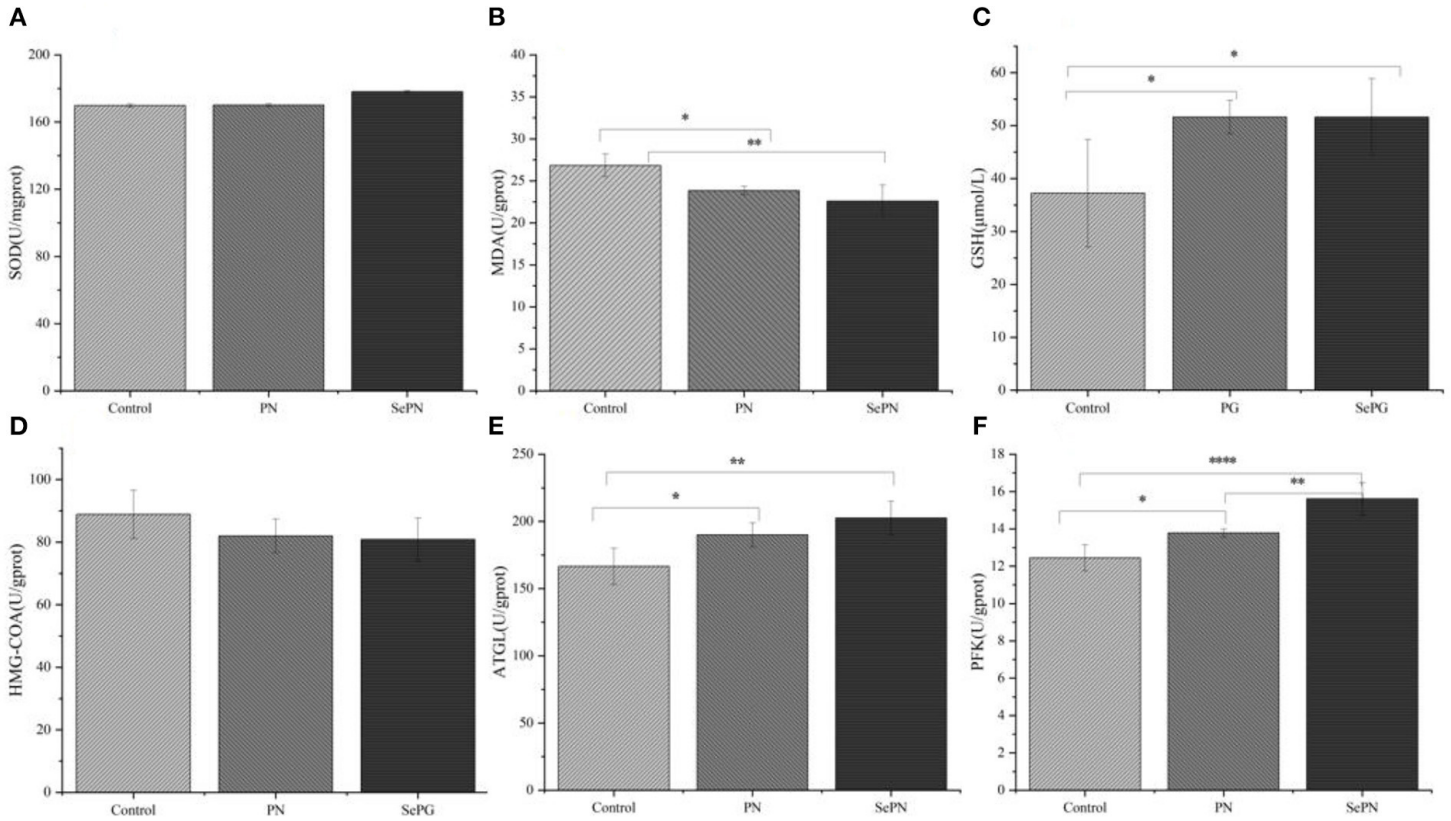
Antioxidant and enzyme activities of glucolipid metabolism in the liver. **(A)** Liver superoxide dismutase (SOD) levels. **(B)** Liver malondialdehyde (MDA) levels. **(C)** Liver glutathione (GSH) levels. **(D–F)** Effects of PN/SePN on liver hydroxymethylglutaryl CoA reductase (HMG-CoAR), adipose triglyceride lipase (ATGL), and phosphofructokinase (PFK). Data are expressed as mean ± SD. *P < 0.05, **P < 0.01, ***P < 0.001, and ****P < 0.0001 compared with the control group (*n* = 5).

After testing the antioxidant stress index of mouse liver, we found that taking PN and SePN could alleviate the oxidative damage caused by reactive oxygen species and reduce MDA levels to some extent (Figure 3B). The treatment groups significantly increased the activity of GSH, but there was no difference between the treatment groups (Figure 3C). What's more, we detected some rate-limiting enzymes of glycolipid metabolism, and we found that compared with the PN group, ingesting SePN could reduce the activity of cholesterol synthase to a certain extent (Figure 3D), increase the activity of ATGL and PFK in glucolipid metabolism (Figures 3E,F). These results showed that compared with PN, SePN could relieve liver injury by improving the ability of antioxidant stress, and protecting the function of glucolipid metabolism of the liver.

### Representative images of H&E staining in colon and liver sections

**Figure 4:**
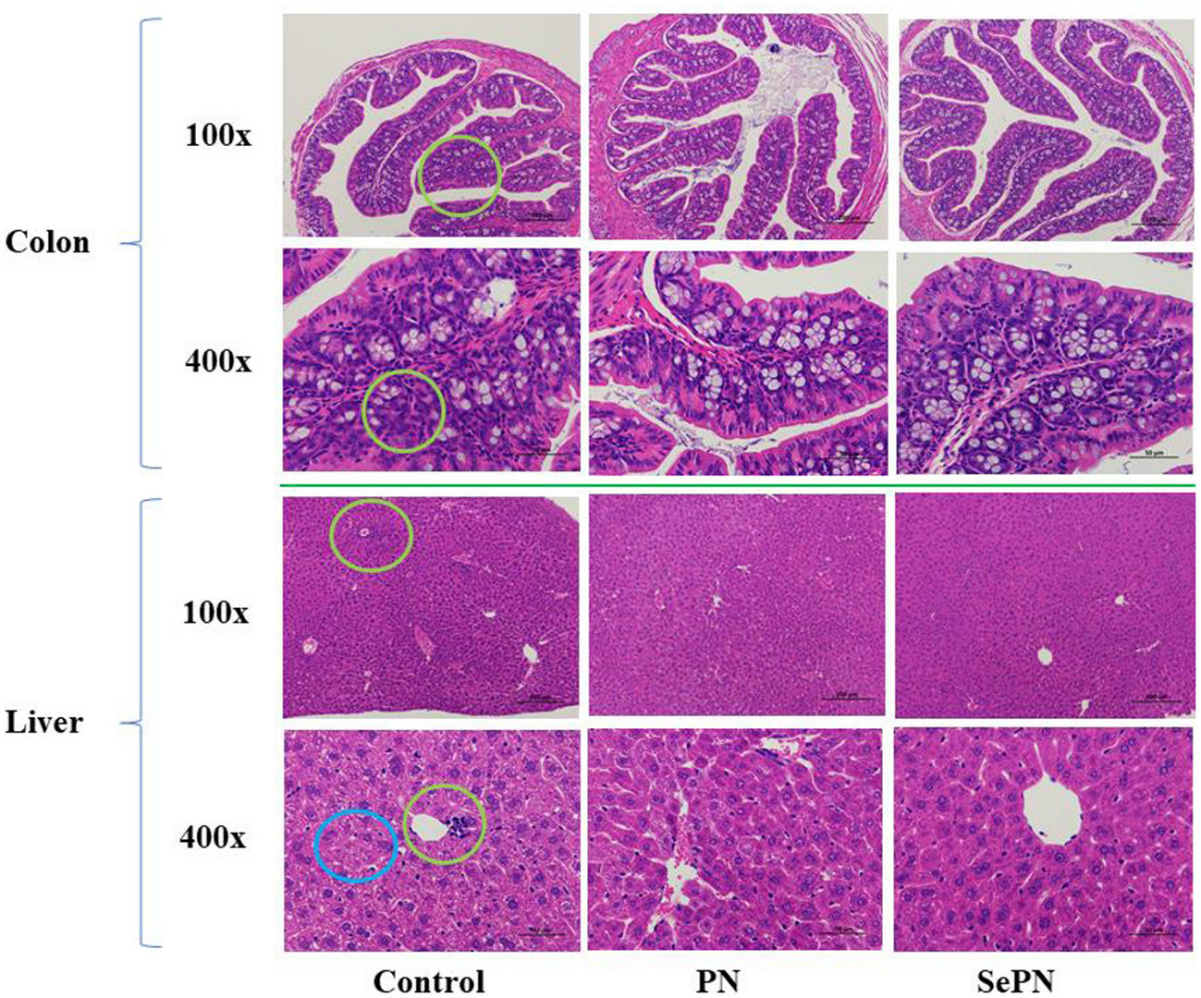
Representative images of H&E staining in colon and liver sections. We use green circles to indicate inflammatory cell infiltration and blue circles to indicate cell vacuolation.

Compared with the H&E staining images of the control group (Figure 4), SePN treatment groups could further increase the number of goblet cells and relieve HFD-induced colon inflammation. Meanwhile, the H&E staining image of the liver indicated that the lymphocytes were infiltrated, activated, and cavitated in the control groups, but the treatment groups could significantly reduce the chronic inflammation hazardous effect to the liver.

### Detection of the expression of lipid metabolism related genes by RT-PCR

**Figure 5:**
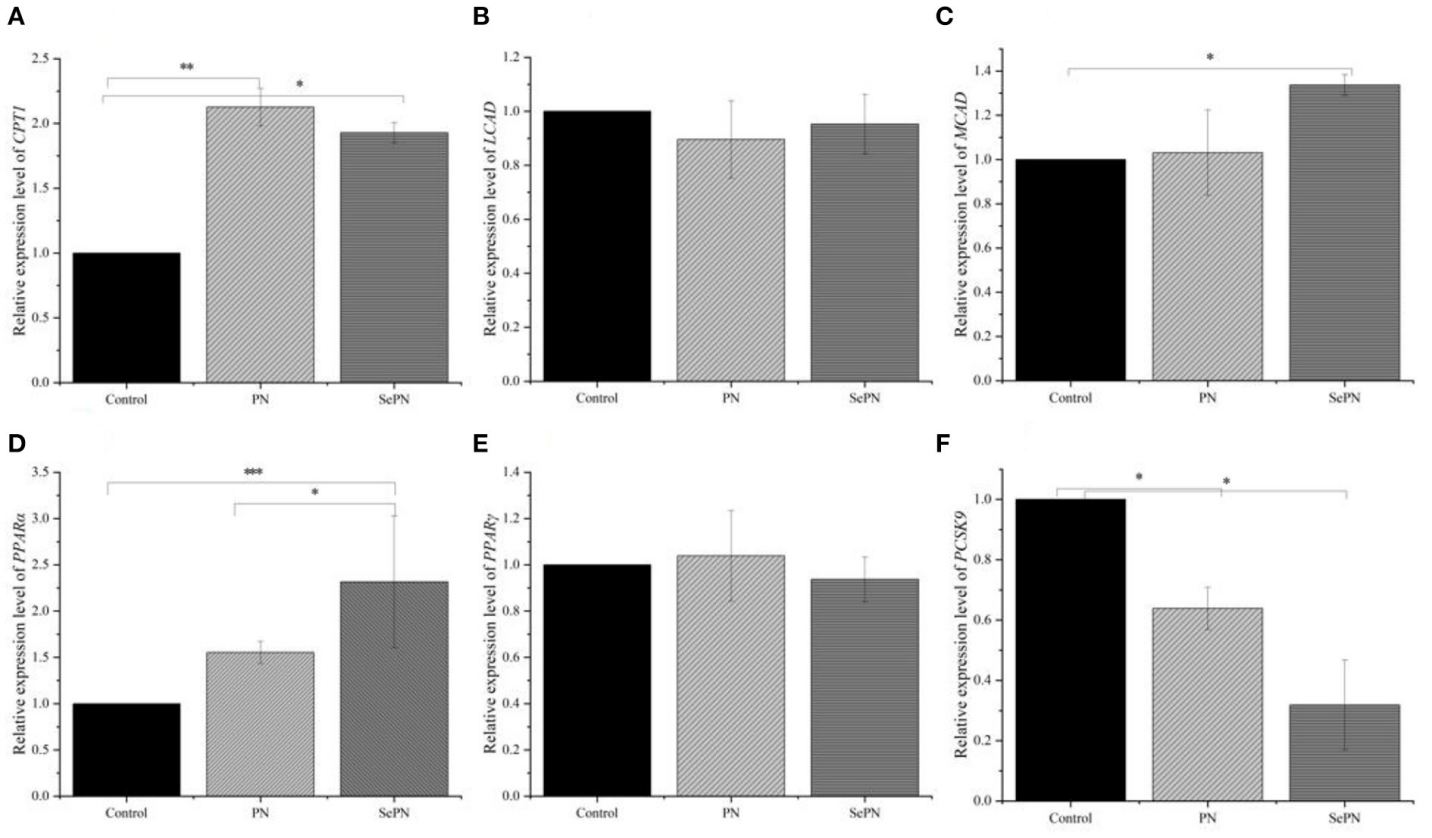
Detection of the expression of glucolipid metabolism related genes by RT-PCR. **(A)**. The relative abundance of mRNA of *CPT1*. **(B,C)** The relative abundance of mRNA of *LCAD* and *MCAD*. **(D,E)** mRNA relative abundance of *PPAR-*α *and PPAR-*γ. **(F)** The relative abundance of mRNA of *PCSK9*. Data are expressed as the mean ± SD. *P < 0.05, **P < 0.01, ***P < 0.001 and compared with the control groups (*n* = 3).

CPT1 is the key enzyme for the entrance of lipid into mitochondria, and oxidative enzymes MCAD, and LCAD will determine the lipid used for the TCA cycle in oxidation. We found that SePN could increase the expression of *CPT1* gene by nearly two times (Figure 5A). Secondly, we detected the expression of *LCAD* and *MCAD*, the other two key genes of fatty acid β-oxidation, and found that the expression of *MCAD* was enhanced, which meant that the ability of medium-chain fatty acid β-oxidation metabolism was enhanced. However, it had no significant effect on the expression level of *LCAD*, which meant that SePN could specifically increase the activity of fatty acid oxidase (Figures 5B,C). *PPAR-*α and *PPAR-*γ play key roles in maintaining glucose and lipid homeostasis by modulating gene expression. Our studies found that SePN significantly up-regulated the expression of *PPAR-*α, but it had no significant effect on *PPAR-*γ expression (Figures 5D,E). In addition, SePN significantly down-regulated the expression of *PCSK9* (Figure 5F).

### Changes of gut microbiota abundance in mice

**Figure 6:**
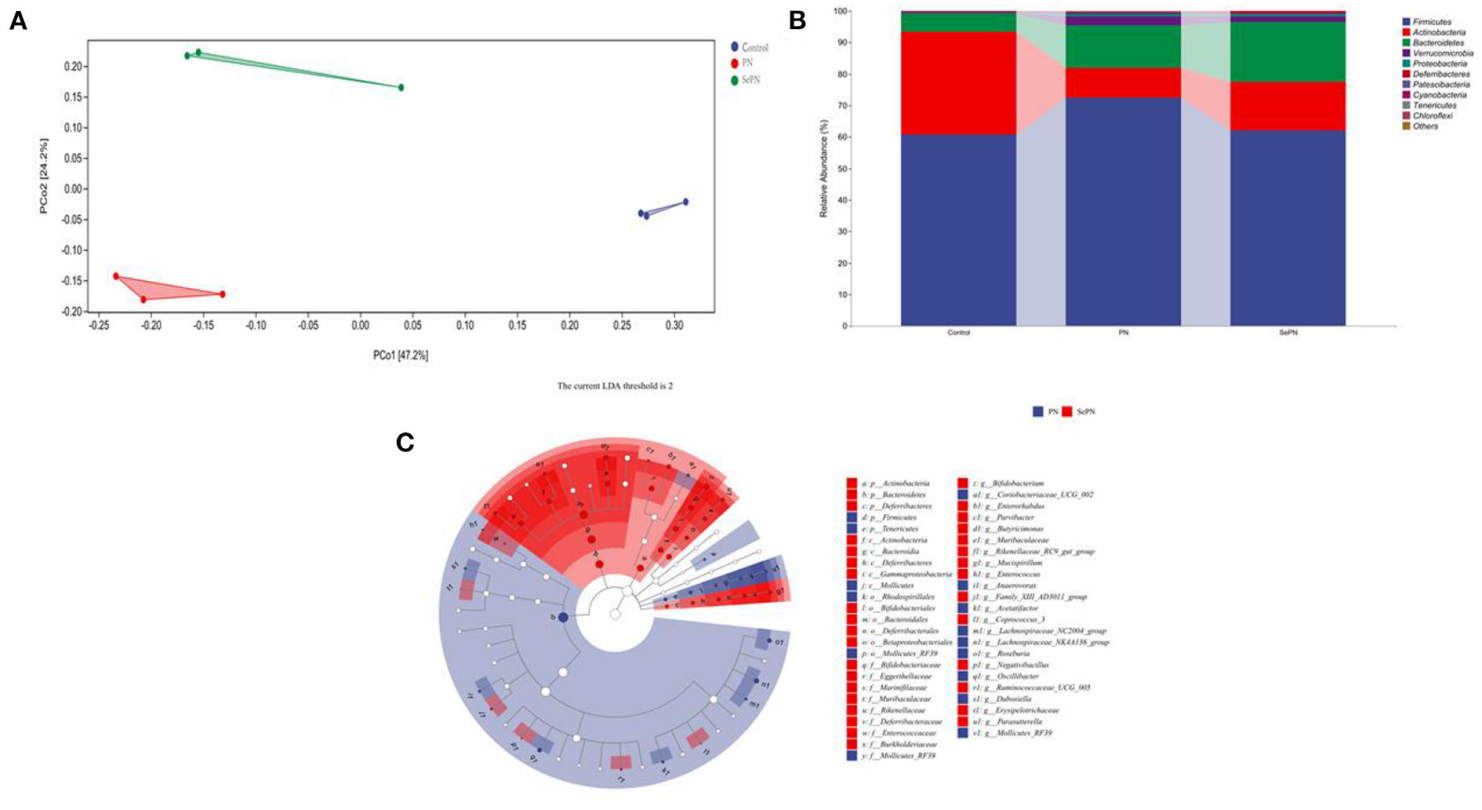
Changes of gut microbiota abundance in mice. **(A)** The principle coordinate analysis (PCoA) plot for the samples in the different groups. **(B**) The gut microbiota composition at the phylum level. **(C)** Taxa with significant differences between groups based on linear discriminant analysis (LDA) effect size.

Sequencing analysis of 16S rRNA gene reveals effects on the composition of gut microbiota. PCoA plots indicated a significant difference in the composition of the gut microbiome between the control and treatment groups (Figure 6A). What's more, population abundance analysis was carried out at the phylum level. The results showed that the gut microbiome was mainly composed of *Firmicutes, Bacteroidetes*, and *Actinobacteria* (Figure 6B). The treatment groups increased the relative abundance of *Bacteroidetes*, which decreased the *Actinobacteria* abundance and *Firmicutes*/*Bacteroides* abundance ratio. To clarify the changes in microbes' abundance among different treatment groups, we performed the LEfSe to find microbes with significant differences (Figure 6C). Notably, in the SePN group, the most significant increase in the abundance of *Bacteroidetes, Butyrimionas, Bifidobacterium*, and *Parasutterella*, and reduce the abundance of *Tenericutes* (Supplementary Figure S1).

## Discussion

Nano-selenium biofortification acts the accumulation of Panax notoginseng saponins (Rb3, Rc, F2, Rb2, and Rf). According to various research, these saponins have the potential to alleviate cardiovascular and cerebrovascular disorders induced by HFD (28–30). Studies showed that selenium deficiency induces Keshan disease, which is characterized by cardiac hypertrophy and increased heart weight (31). We found that the heart weight of mice was reduced after ingesting SePN, which is most likely due to the synergistic effect of Se with saponins. The risk of cardiovascular and cerebrovascular diseases is directly connected to blood sugar and blood lipid contents. The TG, TC, HDL-C, GLU, ALT, and AST levels in the mice serum can directly reflect whether Panax notoginseng protects liver glycolipid metabolism. Our studies discovered that in the treatment groups, TG, TC, and GLU contents declined while HDL-C rose, implying that blood viscosity decreased, but the blood flow velocity improved, lowering the risk of hyperglycemia and hyperlipidemia. However, the activities of ALT and AST in serum did not change considerably, indicating that taking SePN at the prescribed dose every day could not successfully treat the liver impairment produced by long-term HFD.

To further explore the effect of taking SePN on the liver, we detected the related indexes of oxidative stress and glycolipid metabolism. The results showed that the treatment groups might increase the activity of GSH and decrease MDA levels induced by lipid peroxidation, indicating that SePN could protect liver function by improving antioxidation ability. *HMG-CoAR* is involved in cholesterol synthesis and LDL catabolism in serum. The level of *HMG-CoAR* in the treatment groups decreased relatively, but the difference was not statistically significant. It could be inferred that PNS increased the HMG-CoAR activities, inhibited the cholesterol synthesis metabolism of the liver, and promoted the catabolism of LDL, which were beneficial to reduce cholesterol levels in serum. Secondly, the increasing types of saponins (Rb2, etc.) in SePN might not further inhibit cholesterol synthesis metabolism. ATGL and PFK were used as rate-limiting enzymes to chew through TG and GLU, respectively. Compared with the control group, the enzyme activities of ATGL and PFK decreased significantly in the treatment groups. SePN group more efficiently promoted aerobic respiration of GLU and the mobilization of fat. Furthermore, there was a significant positive correlation between PNS contents and ATGL and PFK enzymes, which meant PNS could activate the synthesis of ATGL and PFK.

The study examined the expression level of lipid metabolism-related factors in the liver to further explore the changes in fatty acid metabolism function. *CPT1* is a key factor that promotes the transfer of fatty acids from cell fluid to mitochondrial inner membrane for β-oxidation (32). Inhibition of *CPT1* expression can lead to lipid accumulation and insulin resistance (33, 34). In the liver, peroxisome proliferators activate receptors α and γ (*PPAR-*α*, PPAR-*γ) to regulate the homeostasis of lipid metabolism (35). Among them, *PPAR-*α is involved in regulating the enzyme activity of gluconeogenesis, lipoprotein synthesis and transport determines the capacity of hepatic fatty acid oxidation in the liver (36). *PPAR-*γ plays an important role in adipogenesis, lipid metabolism, insulin sensitivity, and immune regulation (35). The activation of *PPAR-*γ is adipogenic, and the increased expression of *PPAR-*γ in the liver will lead to steatosis (37). MCAD and LCAD catalyze the first step of fatty acid oxidation and determine the lipid used for the TCA cycle (38, 39). Low-density lipoprotein receptor (LDLR) can bind to LDL and reduce LDL contents in serum (40). Subtilisin 9 (*PCSK9*) can negatively regulate the expression of *LDLR*, resulting in hypercholesterolemia (41). Our studies indicated that PNS could affect *PPAR-*α and *PCSK9*-related pathways to reduce fatty acid and LDL levels, and SePN would produce better results. Furthermore, studies indicated that SePN with higher PNS contents could reduce the accumulation of lipids and maintain normal glucolipid metabolism by promoting the utilization of fatty acids, thus reducing the contents of blood sugar and blood lipids.

HFD can significantly decrease the number of goblet cells in the colon, which can secrete mucin to form a mucosal barrier, protect epithelial cells, and reduce the risk of colonic inflammation (42). Compared with PN, SePN could significantly increase the number of goblet cells in the colon to resist endogenous or exogenous stimulation and reduce lymphocyte infiltration and vacuolization in the liver. Considering the increase in antioxidant level, we believed that SePN could protect the liver and reduce the chronic inflammation and metabolic disorder caused by lipid peroxidation, and the mechanisms might be due to the combined effect of high accumulation of PNS and Se.

Furthermore, we further investigated the effects of SePN on gut microbiota. HFD decreases *Bacteroides* levels while increasing the amount of *Firmicutes*, which was a characteristic of the gut microbiota of obese people, according to 16s rRNA sequencing (43). The abundance of *Bacteroides* rose dramatically after the treatment of PN and SePN therapy, but the abundance of *Actinobacteria* declined comparatively. A lower *Firmicutes* / *Bacteroides* ratio indicates a lower risk of obesity (44). Moreover, we used the LEfSe further analyze the differences in gut microbiota composition among groups. Studies showed that *Butyricimonas*, which can produce butyric acid to improve the inflammatory response (43, 45, 46), *Bifidobacterium*, which produces acetic acid to regulate the induction of cholesterol biosynthesis (43, 47), and *Parasutterella*, which is involved in bile acid homeostasis maintenance and cholesterol metabolism (43, 48), increased their abundance after taking SePN. Compared with PN group, taking SePN increased the relative abundance of *Muribaculaceae, Rikenellaceae, Erysipelotrichaceae* (Supplementary Figure S1). At present, studies showed that the increase in the abundance of these microorganisms is negatively correlated with the risk of disease induced by HFD (49–52). Interestingly, some studies showed that dietary selenium supplementation can improve their relative abundance (53, 54). Therefore, the increase of PNS and selenium content jointly regulate the gut microbiota and reduce the negative effects of HFD. These results confirmed that SePN had a better role in regulating glycolipid metabolism and relieving inflammatory reactions. There are significant changes in glucolipid metabolism in mice juvenile to young adulthood. These changes and the potential effects of selenium absorption and transmission regulation of glucolipid metabolism in mice need to be further studied.

## Conclusion

In conclusion, we combined plant and animal experiments together and hoped to fully demonstrate the meaning of the nano-selenium biofortification on Chinese herbal medicine Panax notoginseng by the target determination and 16S rRNA gene sequence analysis. Our study indicated that taking SePN instead of PN might boost antioxidant capacity, glucolipid metabolism enzyme activities, *PPAR*α and *PCSK9* pathway regulation, and gut microbiota improvement linked to glucolipid metabolism, lowering the risk of hyperglycemia, hyperlipidemia, and inflammatory reactions induced by the HFD.

## Data availability statement

The datasets presented in this study can be found in online repositories. The names of the repository/repositories and accession number(s) can be found below: 185824296@qq.com, https://datadryad.org/stash/share/wNEl5d0IV0JZZrwQgHbSDHyBiFaCSbAu2D71_cNlmMU.

## Ethics statement

The animal study was reviewed and approved by Institutional Animal Care Program, China Agricultural University.

## Author contributions

QD planned the study and prepared the manuscript and analyzed the data. SY, WZ, and DL designed the research. QD, YW, ST, and PM carried out experiments. DL, SY, and CZ contributed to the preparation of the manuscript. WZ provided platform and technical support. SZ provided the planting technology of Panax notoginseng and completed the sample collection. CP modified the manuscript and approved the implementation of this experiment. All authors contributed to the article and approved the submitted version.

## Funding

The Major Science and Technology Project in Yunnan Province (202102AE090042, 202105AE160016).

## Conflict of interest

The authors declare that the research was conducted in the absence of any commercial or financial relationships that could be construed as a potential conflict of interest.

## Publisher's note

All claims expressed in this article are solely those of the authors and do not necessarily represent those of their affiliated organizations, or those of the publisher, the editors and the reviewers. Any product that may be evaluated in this article, or claim that may be made by its manufacturer, is not guaranteed or endorsed by the publisher.
