# Supplementary material for: Feeding foliar nano-selenium biofortified panax notoginseng could reduce the occurrence of glycolipid metabolism disorder in mice caused by high-fat diets
Source: Front Nutr. 2022 Aug 24;9:973027. doi: 10.3389/fnut.2022.973027 (PMC9450130; doi:10.3389/fnut.2022.973027)

Supplementary Material

**Panax notoginseng With Nano-selenium Foliar Applications in Field Production Could Reduce the Risk of Glycolipid Metabolism Disorder in Mice Caused by High-fat Diet**

Qinyong Dong^1†^, Sen Yan^1†^, Dong Li^1^, Chunran zhou^1^, Sinuo Tian^1^, Yu Wang^1^, Peijuan Miao^1^, Wentao Zhu^1^, Shusheng Zhu^2^ and Canping Pan^1*^

* Corresponding:

^†^ These authors contributed equally to this work.

E-mail address: canpingp@cau.edu.cn

^1^Innovation Center of Pesticide Research, Department of Applied Chemistry, College of Science, China Agricultural University, Beijing 100193, P. R. China

^2^State Key Laboratory for Conservation and Utilization of Bio-Resources in Yunnan, National Engineering Research Center for Applied Technology of Agricultural Biodiversity, College of Plant Protection, Yunnan Agricultural University, Kunming 650201, Yunnan Province, China

Table S1. The change of medicinal components after nano-Selenium

treatment

| Ginsenoside species | Response | | RSD | | Increase multiple | Average increase multiple |
| --- | --- | --- | --- | --- | --- | --- |
|  | PN | SePN | PN | SePN |  |  |
| R1 | 8.36E+05 | 9.23E+05 | 4.04E+04 | 3.22E+04 | 1.1 | 1.0 |
|  | 9.25E+05 | 8.75E+05 |  |  | 0.9 |  |
|  | 8.92E+05 | 8.91E+05 |  |  | 1.0 |  |
|  | 9.26E+05 | 8.98E+05 |  |  | 1.0 |  |
|  | 9.54E+05 | 9.68E+05 |  |  | 1.0 |  |
| Rf | 6.42E+04 | 6.64E+04 | 1.23E+03 | 8.45E+02 | 1.0 | 1.0 |
|  | 6.64E+04 | 6.67E+04 |  |  | 1.0 |  |
|  | 6.59E+04 | 6.78E+04 |  |  | 1.0 |  |
|  | 6.32E+04 | 6.55E+04 |  |  | 1.0 |  |
|  | 6.37E+04 | 6.76E+04 |  |  | 1.1 |  |
| Rg2 | 1.65E+05 | 1.81E+05 | 1.50E+04 | 1.43E+04 | 1.1 | 1.0 |
|  | 1.77E+05 | 1.71E+05 |  |  | 1.0 |  |
|  | 1.79E+05 | 1.82E+05 |  |  | 1.0 |  |
|  | 1.94E+05 | 1.83E+05 |  |  | 0.9 |  |
|  | 2.09E+05 | 2.13E+05 |  |  | 1.0 |  |
| Rk1 | 3.53E+04 | 3.86E+04 | 2.75E+03 | 2.31E+03 | 1.1 | 1.0 |
|  | 3.79E+04 | 3.63E+04 |  |  | 1.0 |  |
|  | 3.76E+04 | 3.81E+04 |  |  | 1.0 |  |
|  | 3.96E+04 | 3.89E+04 |  |  | 1.0 |  |
|  | 4.35E+04 | 4.33E+04 |  |  | 1.0 |  |
| Rb2 | 1.09E+04 | 4.23E+04 | 2.75E+03 | 2.31E+03 | 3.9 | 3.9 |
|  | 1.12E+04 | 4.01E+04 |  |  | 3.6 |  |
|  | 1.12E+04 | 4.17E+04 |  |  | 3.7 |  |
|  | 1.27E+04 | 4.49E+04 |  |  | 3.6 |  |
|  | 1.25E+04 | 6.18E+04 |  |  | 4.9 |  |
| Rc | 1.44E+04 | 1.51E+04 | 7.22E+02 | 7.96E+03 | 1.0 | 1.1 |
|  | 1.42E+04 | 1.53E+04 |  |  | 1.1 |  |
|  | 1.44E+04 | 1.52E+04 |  |  | 1.1 |  |
|  | 1.60E+04 | 1.64E+04 |  |  | 1.0 |  |
|  | 1.70E+04 | 2.11E+04 |  |  | 1.2 |  |
| Rb3 | 3.28E+04 | 4.22E+04 | 1.12E+03 | 2.28E+03 | 1.3 | 1.4 |
|  | 3.11E+04 | 4.02E+04 |  |  | 1.3 |  |
|  | 3.11E+04 | 4.17E+04 |  |  | 1.3 |  |
|  | 3.54E+04 | 4.47E+04 |  |  | 1.3 |  |
|  | 3.91E+04 | 6.20E+04 |  |  | 1.6 |  |
| Ro | 1.71E+03 | 1.97E+03 | 2.60E+02 | 4.43E+02 | 1.2 | 1.0 |
|  | 2.17E+03 | 2.36E+03 |  |  | 1.1 |  |
|  | 2.00E+03 | 1.85E+03 |  |  | 0.9 |  |
|  | 1.63E+03 | 2.10E+03 |  |  | 1.3 |  |
|  | 1.45E+03 | 1.05E+03 |  |  | 0.7 |  |
| Rd | 9.13E+05 | 1.01E+06 | 2.60E+02 | 4.43E+02 | 1.1 | 1.1 |
|  | 9.54E+05 | 1.01E+06 |  |  | 1.1 |  |
|  | 9.40E+05 | 9.60E+05 |  |  | 1.0 |  |
|  | 9.12E+05 | 1.01E+06 |  |  | 1.1 |  |
|  | 9.04E+05 | 1.04E+06 |  |  | 1.2 |  |
| F2 | 2.35E+04 | 2.87E+04 | 1.64E+03 | 1.88E+03 | 1.2 | 1.3 |
|  | 2.56E+04 | 2.96E+04 |  |  | 1.2 |  |
|  | 2.24E+04 | 2.90E+04 |  |  | 1.3 |  |
|  | 2.30E+04 | 3.32E+04 |  |  | 1.4 |  |
|  | 2.05E+04 | 2.77E+04 |  |  | 1.4 |  |
| Rg1 | 4.32E+06 | 3.78E+06 | 1.64E+03 | 1.88E+03 | 0.9 | 0.9 |
|  | 4.84E+06 | 3.86E+06 |  |  | 0.8 |  |
|  | 5.14E+06 | 3.85E+06 |  |  | 0.7 |  |
|  | 4.98E+06 | 5.01E+06 |  |  | 1.0 |  |
|  | 4.96E+06 | 5.55E+06 |  |  | 1.1 |  |
| Re | 3.19E+04 | 3.31E+04 | 1.64E+03 | 9.75E+02 | 1.0 | 1.0 |
|  | 3.58E+04 | 3.18E+04 |  |  | 0.9 |  |
|  | 3.39E+04 | 3.47E+04 |  |  | 1.0 |  |
|  | 3.57E+04 | 3.41E+04 |  |  | 1.0 |  |
|  | 3.64E+04 | 3.31E+04 |  |  | 0.9 |  |
| Rb1 | 2.67E+05 | 2.83E+05 | 1.64E+03 | 9.75E+02 | 1.1 | 0.9 |
|  | 3.04E+05 | 2.78E+05 |  |  | 0.9 |  |
|  | 2.84E+05 | 2.91E+05 |  |  | 1.0 |  |
|  | 4.00E+05 | 2.84E+05 |  |  | 0.7 |  |
|  | 4.19E+05 | 3.09E+05 |  |  | 0.7 |  |

| *LDL-R* | Forward: GGACCTCAAGATTGGCTACGAG  Reverse: TCGTGGCGATTAGTGAAGAGC |
| --- | --- |
| *PCSK9* | Forward: CCTGCCTTTGTGGTGAAGATGA  Reverse: TGACCCTGCCCTCAATTTCC |
| *PPAR-α* | Forward: TGAAAGATTCGGAAACTGC  Reverse: TTCCTGCGAGTATGACCC |
| *PPAR-γ* | Forward: TCATCTCAGAGGGCCAAGGATTC  Reverse: TGCATTGAACTTCACAGCAAACTCA |
| *LCAD* | Forward: TGCCCTATATTGCGAATTACGG  Reverse: CTATGGCACCGATACATTGC |
| *MCAD* | Forward: CCGAAGAGTTGGCGTATGGG  Reverse: GGGCTCTGTCACACAGTAAGC |
| *β-actin* | Forward: GGCTGTATTCCCCTCCATCG  Reverse: CCAGTTGGTAACAATGCCATGT |
| *CPT1* | Forward: TCTTCTTCCGACAAACCCTGA  Reverse: GAGACGGACACAGATAGCCC |

Table S2. Parameters for saponins analysis by LC-MS/MS

| **Name** | **Precursor(m/z)** | **Product(m/z)** | **Retention time(min)** | **Collision energy (CE)(V)** | **Polarity** |
| --- | --- | --- | --- | --- | --- |
| F_2_ | 783.5 | 621.3 | 5.6 | 40 | Negative |
| R_c_ | 955.6 | 793.5 | 4.22 | 40 | Negative |
| R_1_ | 955.5 | 775.5 | 2.96 | 40 | Positive |
| R_b1_ | 1131.6 | 789.5 | 4.07 | 40 | Positive |
| R_b2_ | 1077.6 | 945.5/783.5 | 4.22 | 40 | Negative |
| R_b3_ | 1077.6 | 945.5/783.5/621.4 | 4.3 | 40 | Negative |
| R_c_ | 1077.6 | 783.5/621.4/459.4 | 4.22 | 40 | Negative |
| R_d_ | 969.6 | 789.5 | 4.45 | 40 | Positive |
| R_e_ | 945.5 | 637.5/475.5 | 3.16 | 40 | Negative |
| R_f_ | 799.5 | 637.5/475.5 | 3.17 | 40 | Negative |
| R_g1_ | 823.5 | 643.4 | 3.14 | 40 | Positive |
| R_g2_ | 783.5 | 637.4/475.4/391.3 | 4.16 | 40 | Negative |
| R_g3_ | 783.5 | 621.5 | 5.59 | 40 | Negative |
| R_h1_ | 823.5 | 643.4 | 3.14 | 40 | Positive |
| Rk_1_ | 765.5 | 441.4 | 4.16 | 40 | Negative |

Table S3. The sequence information of all primer

| *LDL-R* | Forward: GGACCTCAAGATTGGCTACGAG  Reverse: TCGTGGCGATTAGTGAAGAGC |
| --- | --- |
| *PCSK9* | Forward: CCTGCCTTTGTGGTGAAGATGA  Reverse: TGACCCTGCCCTCAATTTCC |
| *PPAR-α* | Forward: TGAAAGATTCGGAAACTGC  Reverse: TTCCTGCGAGTATGACCC |
| *PPAR-γ* | Forward: TCATCTCAGAGGGCCAAGGATTC  Reverse: TGCATTGAACTTCACAGCAAACTCA |
| *LCAD* | Forward: TGCCCTATATTGCGAATTACGG  Reverse: CTATGGCACCGATACATTGC |
| *MCAD* | Forward: CCGAAGAGTTGGCGTATGGG  Reverse: GGGCTCTGTCACACAGTAAGC |
| *β-actin* | Forward: GGCTGTATTCCCCTCCATCG  Reverse: CCAGTTGGTAACAATGCCATGT |
| *CPT1* | Forward: TCTTCTTCCGACAAACCCTGA  Reverse: GAGACGGACACAGATAGCCC |

Figure S1. LDA effect size of treatments


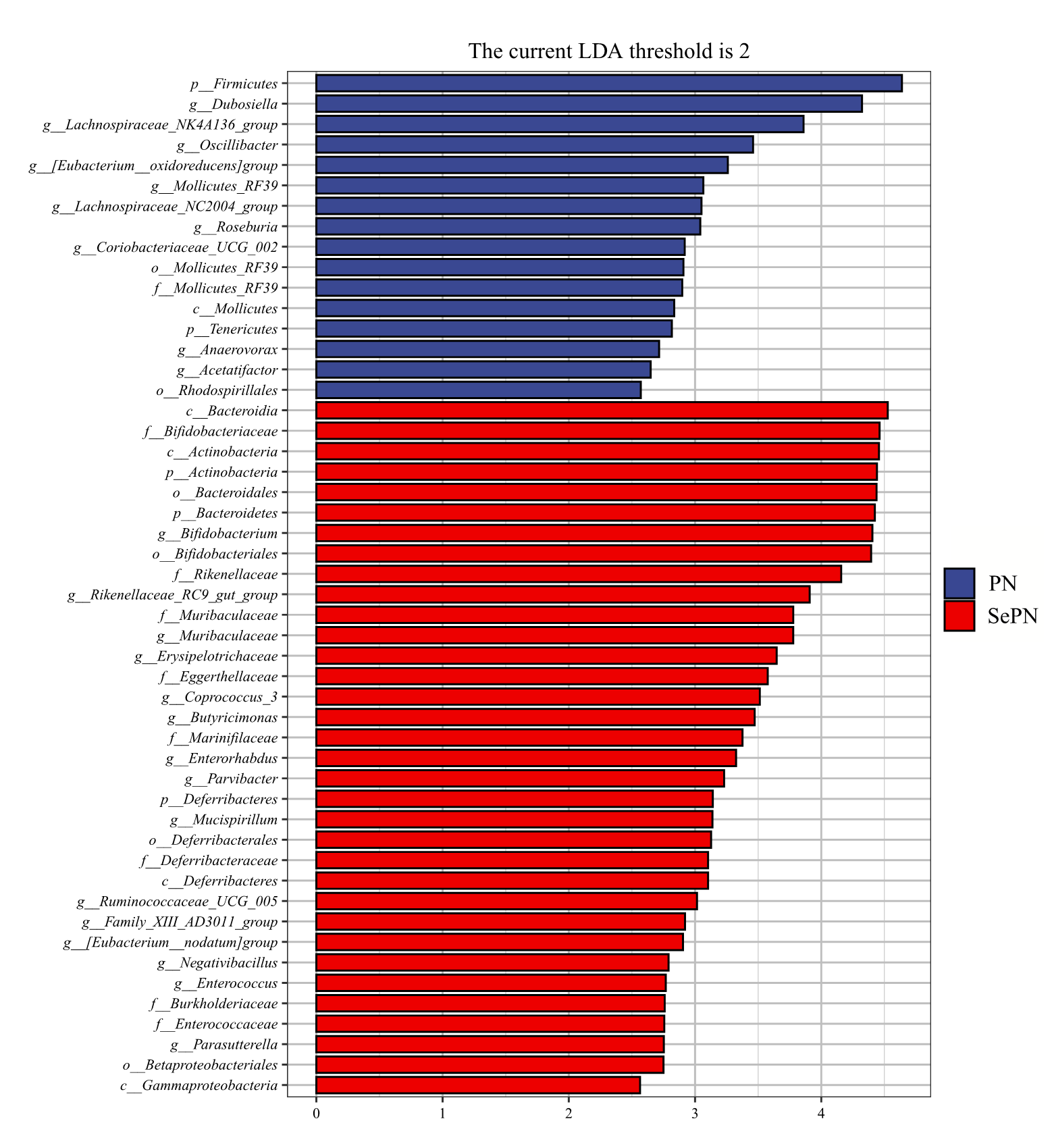

Supplement: Supplementary file 1 [file Data_Sheet_1.docx]
